# A Microfluidic Approach for Inducing Cell Rotation by Means of Hydrodynamic Forces

**DOI:** 10.3390/s16081326

**Published:** 2016-08-19

**Authors:** Stefania Torino, Mario Iodice, Ivo Rendina, Giuseppe Coppola, Ethan Schonbrun

**Affiliations:** 1 Institute for Microelectronics and Microsystems, National Research Council, Naples 80131, Italy; mario.iodice@cnr.it (M.I.); ivo.rendina@cnr.it (I.R.); giuseppe.coppola@cnr.it (G.C.); 2 Rowland Institute at Harvard, Harvard University, 100 E. Land Blvd., Cambridge, MA 02142, USA; schonbrun@rowland.harvard.edu

**Keywords:** microfluidics, rotation, hydrodynamic manipulation

## Abstract

Microfluidic technology allows to realize devices in which cells can be imaged in their three-dimensional shape. However, there are still some limitations in the method, due to the fact that cells follow a straight path while they are flowing in a channel. This can result in a loss in information, since only one side of the cell will be visible. Our work has started from the consideration that if a cell rotates, it is possible to overcome this problem. Several approaches have been proposed for cell manipulation in microfluidics. In our approach, cells are controlled by only taking advantages of hydrodynamic forces. Two different devices have been designed, realized, and tested. The first device induces cell rotation in a plane that is parallel (in-plane) to the observation plane, while the second one induce rotation in a plane perpendicular (out-of-plane) to the observation plane.

## 1. Introduction

In recent years microfluidic technology has attracted the interest of the medical and biology communities. Indeed, the microscale brings significant improvements in cell analysis and characterization. Microfluidics allows building systems in which cells can be imaged while they are flowing in a channel [1,2,3,4]. The main advantage is that biological samples can be observed while they are in their three-dimensional shape. For example, white blood cells (WBCs) and red blood cells (RBCs) can be analyzed in conditions mimicking blood circulation [5]. This is a significant breakthrough, if we consider that biological samples analyzed by means of traditional microscopy are compressed on a glass slide. Even high-performing microscopy systems are affected by this limitation. For instance, let consider scanning confocal microscopy. This technique allows the imaging of cells by different angles of view and, therefore, a 3D reconstruction can be obtained. However, the imaging is still performed on samples prepared in a traditional way, namely cells compressed on a glass slide. Sometimes, in biology research, there is the need of understanding the behavior of complex mechanisms. Knowing how proteins interact with each other, combined with information related to their local concentration in the cell, and with how they modify in time, could give a large improvement in the traditional way in which research in biology is carried out. In addition, volumetric cell imaging could allow identification and characterization of various cells, such as cancer cells, in blood circulation. Although microfluidics can help in developing systems in which cells can be imaged in their three-dimensional shape, on the other hand, the usual way of observing a sample flowing in a microfluidic channel still has a limitation: only one side of each cell will be facing the observer or the imaging point of view, since flowing cells usually follow a straight path. This means that some information can be lost. Imaging flowing cells from different points of view overcomes this limitation. A solution to make this feasible, without moving the experimental setup, is to rotate the cell. Considering the high impact that a technology like this will have in biology research, several methods for cell manipulation in microfluidics have been investigated.

### 1.1. Electrical Manipulation

The most used method for cell manipulation is based on dielectrophoresis (DEP) [6,7]. In the absence of an external electric field, a dielectric particle shows dipoles that are randomly oriented in space. If an external electric field is applied, the dipoles will start to follow the field orientation [8]. Therefore, according to the characteristics of the electric field, the net DEP forces generated on a dielectric particle or cell can be used to move them. Alternating (AC) electric fields have often been used in order to build microfluidic devices for cell manipulation, including translation and rotation. Numerous applications have demonstrated the feasibility of using this approach [9,10]. By engineering the electrodes, their position, size, and shape, the desired rotational effects are achieved. However, most of the proposed devices are characterized by vertical electrodes placed on the channel walls [11]. In this way, the AC field is only able to control the rotation along one single axis. In order to induce a cell rotation along multiple axes, electrodes must be placed both vertically, along the channel walls, and horizontally, on the bottom and/or the top of the channel. Benhal et al. studied this kind of device [12]. However, the required fabrication process is rather complex. In addition, the applied external electric fields could alter the biological sample under analysis and, therefore, compromise the results.

### 1.2. Optical Manipulation

Optical trapping is an established technique for manipulation of cells and biological micro- and nano-molecules. Several years ago it was demonstrated that optical forces can be applied in order to manipulate micro-sized dielectric particles when they are immersed in water or air [13]. Since then, light has been widely used for trapping micro-objects in different fields of applications, ranging from physics to biology [14,15,16,17,18]. The most common adopted method is based on a single-beam gradient force, most commonly known as optical tweezers (OT). In addition to this configuration, an approach based on a two beam optical trap has been widely exploited [19]. When two counter-propagating laser beams are simultaneously applied, micro-objects can be trapped in the area where the forces induced from the two beams are balanced. Kolb et al. [20] showed how a dual beam optical trap and the effect of a Poiseuille flow in a microfluidic channel can be adopted for obtaining a high-throughput cell rotation. The dual beam configuration has also been adopted by other research groups for control cell orientation and rotation. Dasgupta and Kreysing [21,22] have shown how to manipulate RBCs and cancer cells by choosing the right properties for the laser beam.

### 1.3. Acoustophoresis

Acoustophoresis standss for *phoresis*, migration, and *acousto*, sound waves, so the term refers to the possibility to move, guide, or manipulate objects using acoustic waves. Recently, a novel term has been created, *acoustofluidics* [23], which refers to the use of acoustophoresis in microfluidics. At first acoustic waves were used for the realization of separation and filtering devices [24,25,26,27,28]. Later, we find the combination of the acoustic effects with optical trapping based on a dual beam configuration for stretching biological objects [29]. Li et al. [30] realized a lab-on-chip platform in which inflammatory cells are extracted from liquefied human sputum samples by means of acoustic forces. In 2016, Ahmed and co-workers [31] showed an acoustic-based method for rotation of micro-objects. In their device, microbubbles are trapped within predefined sidewall microcavities, then they are driven into oscillatory motion through an acoustic field, generating microvortices, which are utilized for rotation of colloids, cells, and entire organisms.

### 1.4. Magnetofluidic Trapping

Magnetic field has also been investigated as a possible approach for cell or particle manipulation [32]. As well as the other methods based on the application of external forces, magnetic elements for field generation have to be inserted into the device. This leads to an increase of the complexity of the fabrication process. Moreover, in most of the cases a pre-treatment of the biological sample is required [33], thus possible alterations of the sample can be induced [34]. To overcome this limit, Hejazian and Nguyen [35] have recently showed how diluted ferrofluid and permanent magnets can be used for the size-selective concentration of non-magnetic particles. Two arrays of permanent magnets placed at the top and at the bottom of the channel, respectively, allows the generation of multiple capture zones and the interaction between magnetic forces and hydrodynamic forces allows the device to operate in different regimes suitable for the concentration of non-magnetic particles with small differences in size.

### 1.5. Hydrodynamic Manipulation

Considering limitations and complexities introduced by the approaches based on the use of external forces, in this work the possibility to rotate cells by only taking advantage of hydrodynamic effects has been studied. Several examples of microfluidic devices based on hydrodynamic manipulation [36,37] have already been proposed. For example, single cell and microparticle rotation has been achieved using the concept of a microvortex, which relies on the creation of a recirculating flow profile [38]. The microvortices are generated by fabricating channels with diamond-shaped side chambers [15,39]. Hagiwara et al. [40] proposed a different approach in which cells are manipulated by locally controlling the fluid streamlines through a micro-tool introduced inside the channel.

High-frequency oscillation of this microtool is induced, and by tuning the oscillation parameters, such as the amplitude and phase of the oscillation, the streamlines can be controlled. Authors have demonstrated the feasibility to manipulate bovine oocytes position and rotation within the device. A similar approach that can be considered is the one proposed by Lutz et al. [41]. They used *hydrodynamic tweezers* for suspending cells using the hydrodynamic traps created by low audible frequency oscillations of the fluid medium in a microchannel containing a single fixed cylinder. The only requirement is that cells have to be less or more dense than the surrounding medium. In our work we propose a passive manipulation method based on hydrodynamic effects. The device does not require any additional elements, by simplifying the device fabrication and at the same time avoiding undesired effects on cells. The operating principle is based on the consideration that in a pressure driven flow, the velocity follows the Hagen-Poiseuille law [42] and, therefore, it is characterized by a parabolic profile. The velocity gradient generated along a channel is also the basis of inertial focusing [43,44], in which the inertia of the fluid around the particles is used to force them into a confined stream. However, there is a significant difference in our work, since our fluid velocity is very slow compared to the inertial regime, and, therefore, inertial effects can be neglected. In our regime the Reynolds number *Re << 1*. The characteristic in which we are interested is that in a Poiseuille profile, the fluid velocity has its maximum value at the center of the channel, and it reaches the lowest values close to the channel walls. Moreover, in a close channel, the previous statement is true on all four walls and, therefore, the velocity profile can be described as a paraboloid. Based on this assumption, we designed two microfluidic devices, in which cell rotation is achieved: (1) in the in-plane direction (plane parallel to the observation plane) by focusing the cells close to one of the channel walls; and (2) in the out-of-plane direction (plane perpendicular to the observation plane) by focusing the cells at the bottom of the channel. In both of these configurations, the two opposite sides of the cell, the one close to the wall and the other closer to the channel center, will experience two different flow velocity values. As a consequence, a torque will act on the cell and a rotation will be induced. In order to verify the correct operation of the two devices, we used yeast cells (*Saccharomyces cerevisiae*), since they present a bud formation when they are in the duplication phase. This asymmetrical shape allowed us to follow the cell rotation in the post-processing imaging analysis.

## 2. Theoretical Background

**Figure 1:**
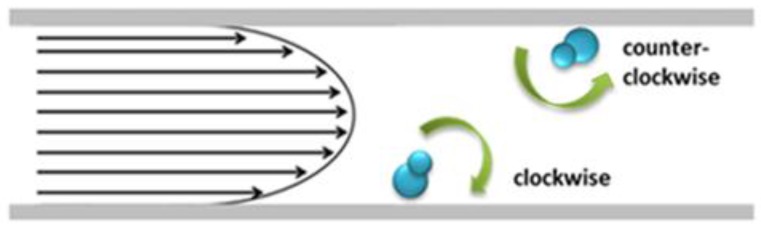
The cartoon explains the rotation effect induced by the parabolic velocity profile, when objects are close to the channel walls.

The Hagen-Poiseuille equation is the physical law that relates the pressure drop ΔP in a fluid flowing through a long pipe with its volumetric flow rate Q :

(1) $$\Delta P=R_{hyd}\;Q$$

where $R_{hyd}$ is the hydraulic resistance of the channel. The main assumptions of the equation are that the fluid is viscous and incompressible, and the flow is laminar. These conditions are completely satisfied in microfluidics [45]. To proceed to find the analytic solution for the velocity, the channel cross-section has to be fixed. The general solution for the flow in a channel with a rectangular cross-section is given by the below equations, for the velocity:

(2) $$u\left(y,z\right)=\;\frac{16h^{3}}{\mu \pi^{3}}\;\left(-\frac{dp}{dx}\right)\overset{\infty }{\underset{i=1,3,5\ldots }{\sum }}{\left(-1\right)}^{\left(i-1\right)/2}\left[1-\frac{cosh\left(\frac{i\pi z}{2h}\right)}{cosh\left(\frac{i\pi w}{2h}\right)}\right]\frac{cos\left(\frac{i\pi y}{2h}\right)}{i^{3}}\;$$

and for the flow rate:

(3) $$Q=\frac{4wh^{3}}{3\mu }\;\left(-\frac{dp}{dx}\right)\left[1-\frac{192h}{\pi^{5}w}\;\overset{\infty }{\underset{i=1,3,5\ldots }{\sum }}\frac{tanh\left(\frac{i\pi w}{2h}\right)}{i^{5}}\right]$$

where u(y,z) is the velocity, dpdx is the pressure drop across the channel, h and w the height and the width of the channel cross-section, respectively. The analytic solution for the velocity says that a Hagen-Poiseuille flow is characterized by a parabolic velocity profile, in which the velocity of flow in the center of the channel is greater than that toward the outer walls (Figure 1). Due to the parabolic velocity profile, if a cell is close to the wall its surface will be affected by different velocity values. In particular, the cell side closer to the center of the channel is affected by a higher velocity than the side closer to the wall. The couple of forces acting on the cell generates a torque, and so a rotation is induced. The components of the torque vector $\bar{T}$ can be expressed as follows [46,47]:

(4) $$T_{x^{\prime }}=\frac{16\pi \mu a_{p}^{3}\beta }{3\left(\beta_{0}+\;\beta^{2}\gamma_{0}\right)}\;\left[\left(1-\beta^{2}\right)D_{z^{\prime }y^{\prime }}+\;\left(1+\beta^{2}\right)\left(W_{z^{\prime }y^{\prime }}-\omega_{x^{\prime }}\right)\right]$$

(5) $$T_{y^{\prime }}=\frac{16\pi \mu a_{p}^{3}\beta }{3\left(\alpha_{0}+\;\beta^{2}\gamma_{0}\right)}\;\left[\left(\beta^{2}-1\right)D_{x^{\prime }z^{\prime }}+\;\left(1+\beta^{2}\right)\left(W_{x^{\prime }z^{\prime }}-\omega_{y^{\prime }}\right)\right]$$

(6) $$T_{z^{\prime }}=\;\frac{32\pi \mu a_{p}^{3}\beta }{3\left(\alpha_{0}+\;\beta_{0}\right)}\;\left[W_{y^{\prime }x^{\prime }}-\omega_{z^{\prime }}\right]$$

where

- $x^{\prime },y^{\prime },z^{\prime }$ the particle coordinate system with its origin being at the particle mass center and its axes being the principal axes x, y, and z.
- $D_{i,j}$ the deformation rate tensor , $W_{i,j}$ the spin rate tensor, and defined as follows:
  (7) $${\left[D\right]}_{x^{\prime }y^{\prime }z^{\prime }}=\;\frac{1}{2}\;{\left[\nabla \vec{v_{p}}+\;{\left(\vec{v_{p}}\right)}^{T}\;\right]}_{x^{\prime }y^{\prime }z^{\prime }}$$
  (8) $${\left[W\right]}_{x^{\prime }y^{\prime }z^{\prime }}=\;\frac{1}{2}\;{\left[\nabla \vec{v_{p}}-\;{\left(\vec{v_{p}}\right)}^{T}\;\right]}_{x^{\prime }y^{\prime }z^{\prime }}$$
- $a_{p}$ and $v_{p}$ are, respectively, the minor axis and the linear velocity of the ellipsoidal particle.
- $\beta =\frac{b_{p}}{a_{p}}$, the ratio between the major and minor axes of the ellipsoidal particle, and from this definition $\alpha_{0}$, $\beta_{0}$, and $\gamma_{0}$:
  (9) α0= β0= β2β2−1+ β(β2−1)32ln[β−β2−1β+β2−1]
  (10) γ0= −2β2−1− β(β2−1)32ln[β−β2−1β+β2−1]
- The angular velocity components are:
  (11) $$\omega_{x^{\prime }}=\;\frac{\partial \psi }{\partial t}+\frac{\partial \phi }{\partial t}\cos\theta ,\;\omega_{y^{\prime }}=\;\frac{\partial \theta }{\partial t}\cos\psi +\frac{\partial \phi }{\partial t}\;\sin\theta \sin\psi ,\;\omega_{z^{\prime }}=\;\frac{\partial \phi }{\partial t}\sin\theta \cos\psi -\frac{\partial \theta }{\partial t}\;\sin\psi$$
  with θ, ϕ, ψ the Euler’s angles [48].

## 3. Materials and Methods

### 3.1. Numerical Simulation Analysis

**Figure 2:**
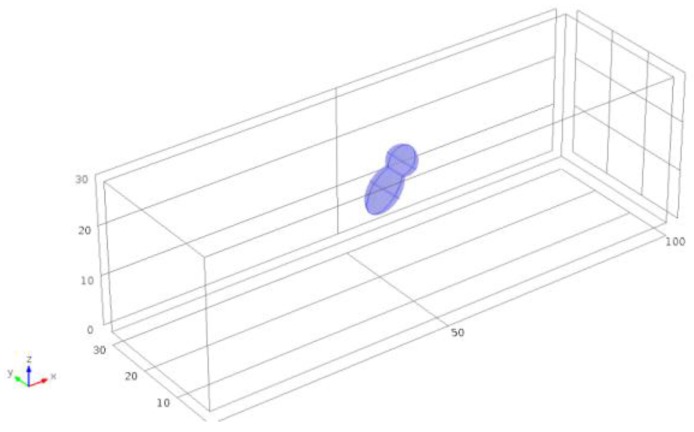
Design of the geometry used for the numerical simulation analysis.

Numerical simulations were performed by COMSOL Multiphysics (COMSOL, Inc., MA, USA), in order to verify the rotation effects induced in the two designed geometries. An asymmetrical object was placed in a channel with a cross-section of 30 μm × 30 μm (Figure 2). We were able to show that the angular velocity of the micro-object depends upon the occupied position across the channel. For simulating the in-plane rotation, the asymmetrical object was placed at five different positions along the *y* direction, whereas the vertical position *z* was fixed at the half of the channel height.

**Figure 3:**
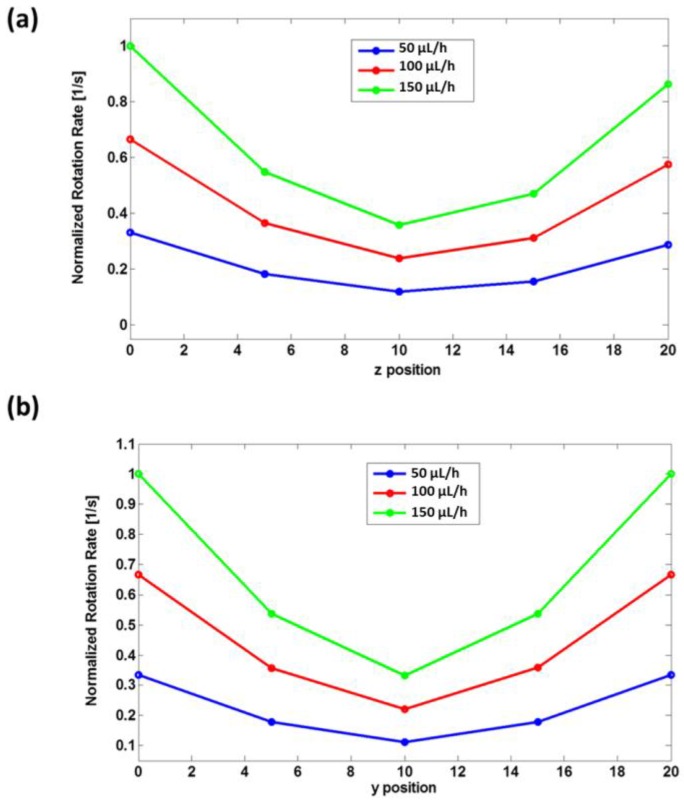
Numerical simulation results. The rotation rate (number of rotation/s) as a function of (**a**) the y horizontal position and (**b**) the vertical position across the channel. Three studies were performed by setting different values for the flow-rate of the fluid at the inlet.

**Figure 4:**
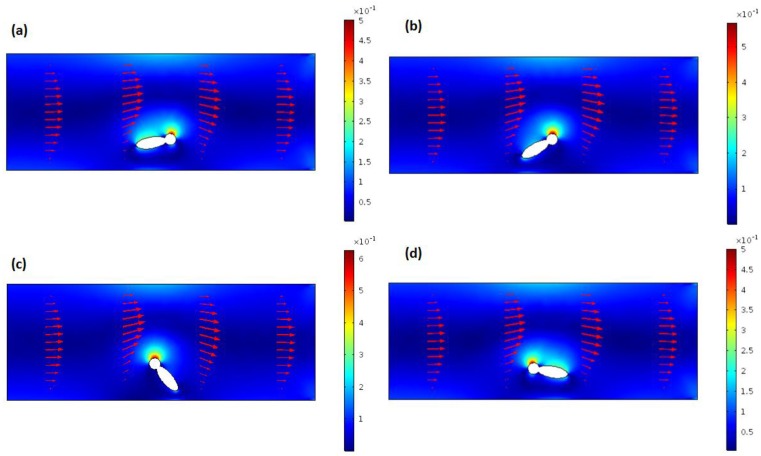
The image shows four of the different angular orientation for the ellipse used for the numerical simulation analysis: (**a**) 10°; (**b**) 30°; (**c**) 120° and (**d**) 170°. The color-map refers to the vorticity field magnitude (lowest magnitude in dark blue, higher magnitude in red).

**Figure 5:**
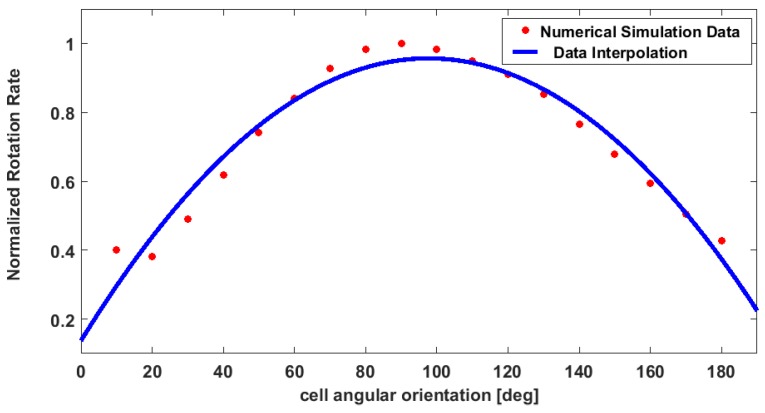
Numerical simulation data. Normalized rotation rate for an ellipse at different angular position respects to the fluid flow direction. The 0° refers to the ellipse in a horizontal position, with the major axis parallel to the fluid flow direction. As expected, when it is in this position, the rotation rate is low, and it increases when it gets close to the vertical position, with the major axis perpendicular to the fluid flow direction.

Then, a similar approach was used in order to simulate the operation of the out-of-plane rotator. In this case the target object was placed at five different positions along the *z* direction, with the *y* position fixed. Water, as defined in the COMSOL Multiphysics library, was set as the bulk material. The flow rate was set to 50, 100, and 150 μL/h. The results are reported in Figure 3a for the in-plane and Figure 3b for the out-of-plane rotator. The data are normalized to the maximum value of the rotation rate. The two graphs show that the rotation rate reaches the minimum value when the target object occupies a position close to the center of the channel. In addition, a 2D numerical analysis has been performed in order to analyze the rotation effect when the object occupies different angular position respect to the fluid flow direction. The simulation has been done by placing the asymmetrical object in a channel with a width of 30 µm. Then, the rotation rate (number of rotation per second) was calculated by changing the orientation of the ellipse from 0° (ellipse with the major axes parallel to the direction of the fluid) to 170°, with a step of 10° (see Figure 4). The rotation rate resulted to be higher when the ellipse is in a vertical position, when the major axis is perpendicular to the fluid flow, and it gets lower when it is in a 0° position. This is what we expected, since when the ellipse is in a vertical position the two opposite sides will experience a much higher velocity gradient. These data are shown in the graph in Figure 5, where the values for the rotation rate are plotted as function of the angular orientation.

### 3.2. Design and Fabrication

**Figure 6:**
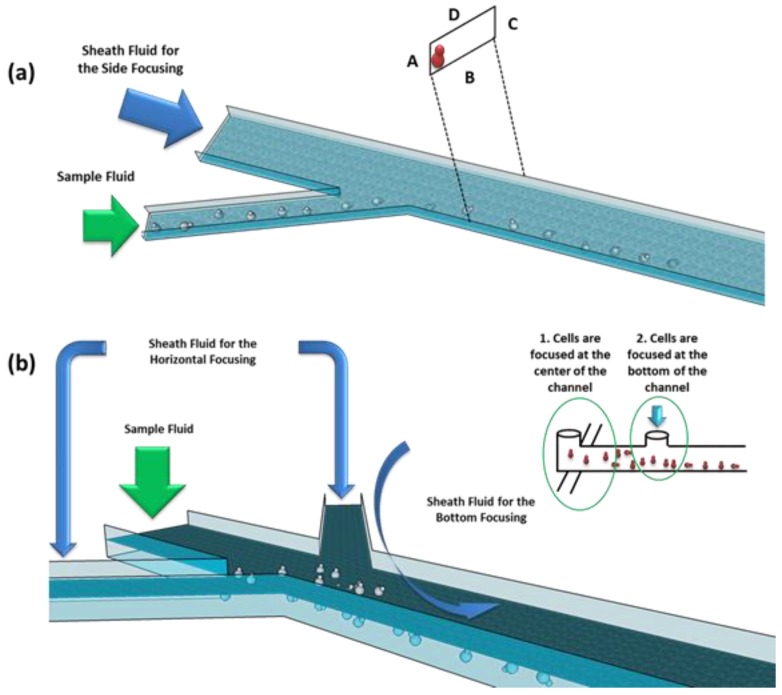
Schematic of the realized devices for the (**a**) in-plane and (**b**) out-of-plane hydrodynamic rotation.

In our devices, hydrodynamic focusing is used in order to confine particles to the channel walls. For the in-plane rotator (Figure 6a), cells coming from a lateral channel are confined at the wall due to a sheath fluid confinement effect. The width of the sample stream being laterally confined depends upon the ratio between the sheath and the sample fluid. The width of the central channel is 30 μm and the lateral ones is 20 μm. Both channels are 10 μm high. For the out-of-plane rotator (Figure 6b), first, cells are confined at the center of the channel, by means of a sheath fluid coming from two lateral channels. After that, an additional sheath fluid coming from the top side is used to confine cells on the bottom of the channel. The height of the main channel was set to about 30 μm.

Both microfluidic devices were realized using the standard polydimethylsiloxane (PDMS) soft-lithography technique. First, a photolithography process was performed in order to obtain a negative master mold of the designed patterns. For the in-plane rotation device, SU8-3010 negative photoresist was spun at 3000 rpm, in order to get a resist thickness of about 10 µm. For the out-of-plane device, SU8-3050 negative photoresist was used during the photolithography process in order to obtain a channel thickness of about 30 µm. A 10:1 mixture of PDMS oligomer and crosslinking agent (Sylgard 184, Dow Corning Corporation), was poured onto the master mold. The polymer was cured in the oven at 80 °C for 2 h. The cured PDMS was gently peeled off, cut, and holes for the inlet and the outlet were performed. The permanent sealing with a thin glass substrate was obtained through oxygen plasma treatment of both surfaces.

### 3.3. Experimental Setup

**Figure 7:**
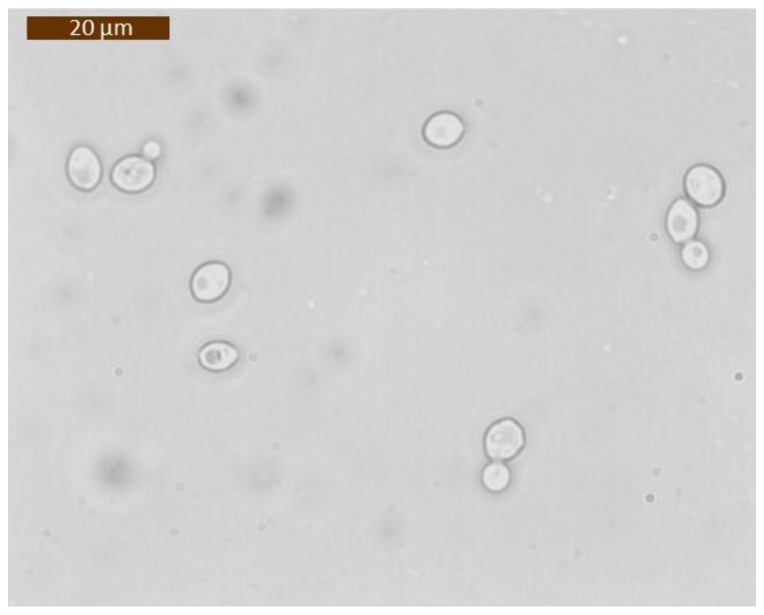
Microscopy image of the *Saccharomyces cerevisiae* cells used for the experimental testing of the microfluidic devices.

The hydrodynamic rotation was experimentally evaluated by means of yeast cells, *Saccharomyces cerevisiae*. *S. cerevisiae* is a small, single-celled fungus and, thus, like other fungi, it has a rigid cell wall [49]. Due to its asymmetrical shape, this cell is ideal to study the rotational effect. Indeed, yeasts, divide by forming a small bud that grows steadily until it separates from the mother cell. A microscopy image of the biological sample used in the experiment is showed in Figure 7. The sample fluid was prepared by mixing 50 µL of a yeast cell solution with 250 µL of a buffer solution. For both of the experiments data were acquired by using the following optical image setup: a high-speed camera (Teledyne DALSA Inc., Waterloo, ON, Canada) was used in order to acquire data at a frame rate feasible with the cell rotational velocity; a white LED was used for illumination; and syringe pumps were used for controlling the sample and sheath flow rates.

## 4. Results

**Figure 8:**
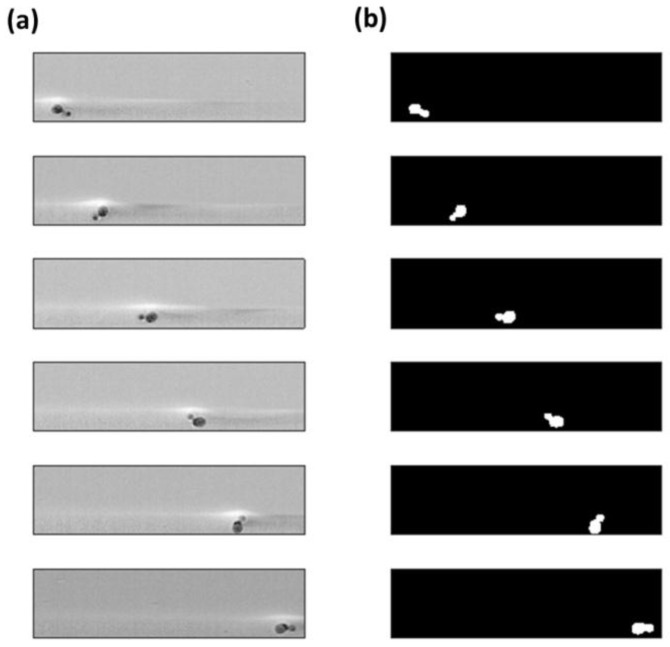
In-plane rotation device: (**a**) In the six consecutive frames, a cell close to the channel wall is induced in rotation; (**b**) The same frames are converted in binary images.

Image processing was performed by Matlab (the MathWorks, Inc., Natick, MA, USA). The induced rotation was estimated by analyzing the acquired videos. Figure 8a shows five consecutive frames concerning the in-plane rotator device. The rotational effect on the cell flowing close to the channel wall is observed. As expected, the cell rotates clockwise, since its upper side experiences a velocity higher than the opposite side close to the channel wall. The binarized images of the same frames, obtained by means of a Matlab code, are reported in Figure 8b. The binary images allowed us to get more information about the cell angular orientation and the occupied position (*x, y*) at each frame.

**Figure 9:**
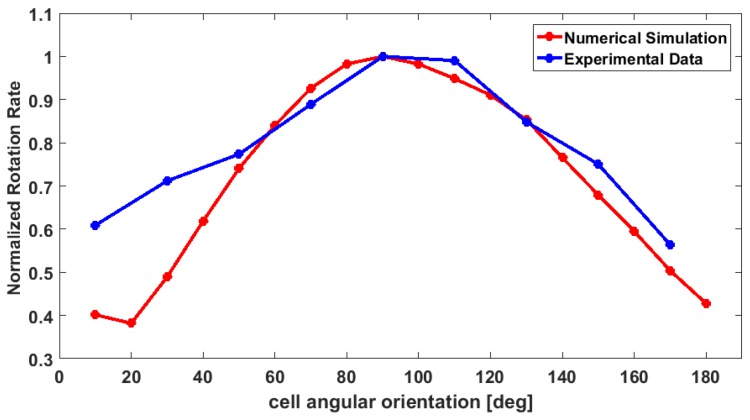
The plot shows the rotation rate (number of rotation/s) in relation to the cell angular orientation both for the experimental and simulation data.

The graph in Figure 9 reports the rotation rate as a function of the cell angular orientation. The experimental data are compared with the data obtained from the numerical simulations (see Figure 5). As we expected, when a cell occupies a position at 60°–140° respect to the fluid direction, the rotation rate reaches the highest values. This can be explained by considering that if the cell is in a vertical position, the cell surface will experience a higher velocity gradient. For the opposite reason, the angular velocity decreases when the cells occupy a horizontal position, which is the situation when a cell angular orientation is close to 0° and 180°). Therefore, the trend of the curve experimentally obtained is in agreement with the one obtained by COMSOL. However, it is evident that a main difference between the two curves exists: the experimental results show a higher rotation rate when the angular orientation gets close to 0°/180°. This behavior can be explained considering that numerical analysis simulates an ideal condition. Indeed, the flow rate is kept constant, without considering any variation. In experimental condition, small variations of the flow rate can accidently occur. In addition, in our model we are not taking into consideration the interaction between the object and fluid, and the object and the channel wall.

**Figure 10:**
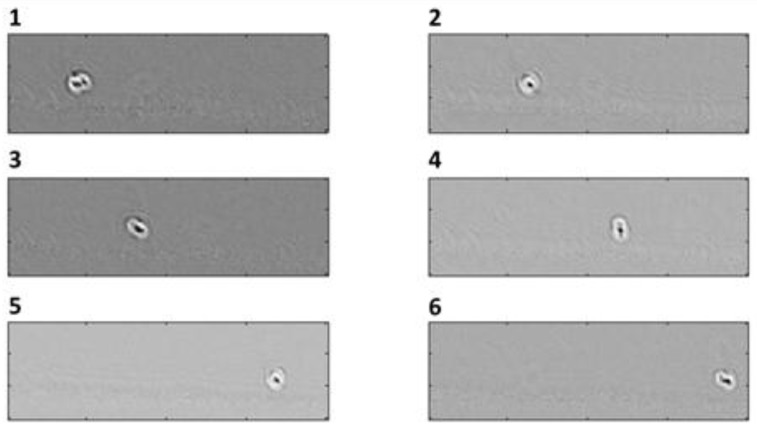
Out-of-plane rotation device. In the six consecutive frames, a cell close to the bottom of the channel is induced in rotation.

In addition to the in-plane rotator, we were able to experimentally demonstrate the correct operation of the out-of-plane rotator device. By analyzing the acquired videos, we observed that cells focused at the bottom of the channel start to rotate due to the hydrodynamic effects. Figure 10 shows six consecutive frames of the collected data. In the first frame a cell and its bud are both visible, whilst in the second one the cell looks as if it were round, i.e., without a bud. This means that the cell is rotating in a plane perpendicular to the imaging plane.

## 5. Conclusions

In this paper, a microfluidic approach for inducing cell rotation by only taking advantage of hydrodynamic forces has been presented. The motivation for this work is related to the fact that cells flowing in a microchannel usually follow a straight path. This means that if the imaging point of view is fixed, only one side of a cell will be visible. Therefore, there will be a loss in information. One possible solution to overcome this problem, without moving the imaging set-up, is to rotate the cells. In order to study this possibility, several research groups have proposed different methods for rotating cells by meaning of different approaches. It has been shown that biological samples can be manipulated by means of optical forces, acoustic waves, magnetic fields, and so on. Moreover, one interesting research field is the one related to manipulation of cells by means of hydrodynamic forces. In this approach no external forces or fields are required. The two main advantages are that no additional elements have to be introduced into the device, and there are any undesired effects induced on the sample under analysis. In this work, two devices have been designed, realized, and tested. In the two different configurations cells are rotated in a plane parallel (in-plane) and perpendicular (out-of-plane) to the imaging plane, respectively. This is achieved by focusing the sample fluid to one of the lateral channel wall, for the in-plane rotation, and to the bottom of the channel, for the out-of-plane rotation. Numerical simulations have been performed in order to show the relation between the rotation rate and (1) the position of the cell across the channel; (2) the angular orientation of the cell respect to the fluid flow direction. The simulation results showed to be in agreement with the experimental data. However, some differences in the simulated and experimental trends are present. These differences have motivated discussion in the results section, and they are mainly due to the fact that the simulation does not consider the interaction between the object and the channel. An improvement in our work could be done by performing numerical analysis by using the COMSOL moving mesh interface. In this way it could simulate the rotation of objects having different shapes, also considering their interactions with the fluid.

In conclusions, in this work we have demonstrated the feasibility of a novel microfluidic approach for rotating cells by means of hydrodynamic forces. The method does not require external forces, by avoiding in this way complexity in the fabrication process, and also undesired effects on the biological sample under analysis.

## References

[1] Huh D., Gu W., Kamotani Y., Grotberg J.B., Takayama S. (2005). Microfluidics for flow cytometric analysis of cells and particles. Physiol. Meas..

[2] Simonnet C., Groisman A. (2006). High-throughput and high-Resolution flow cytometry in molded microfluidic devices. Anal. Chem..

[3] Watkins N., Venkatesan B., Tone M., Rodriguez W., Bashir R. (2009). A robust electrical micro-cytometer with 3-dimensional hydrofocusing. Lab Chip.

[4] Mao X., Nawaz A.A., Lin S.C., Lapsley M.I., Zhao Y., McCoy J.P., El-Deiry W.S., Huang T.J. (2012). An integrated, multiparametric flow cytometry chip using “microfluidic drifting” based three-dimensional hydrodynamic focusing. Biomicrofluidics.

[5] Schonbrun E., Malka R., Di Caprio G., Schaak D., Higgins J.M. (2009). Quantitative absorption cytometry for measuring red blood cell hemoglobin mass and volume. Cytom. Part A.

[6] Han S.I., Joo Y.D., Han K.H. (2013). An electrorotation technique for measuring the dielectric properties of cells with simultaneous use of negative quadrupolar dielectrophoresis and electrorotation. Analyst.

[7] Chau L.H., Liang W., Cheung F.W.K., Liu W.K., Li W.J., Chen S.C., Lee G.B. (2013). Self-rotation of cells in an irrotational AC E-field in an opto-electrokinetics chip. PLoS ONE.

[8] Tabeling P. (2005). Introduction to Microfluidics.

[9] Pethig R. (1996). Dielectrophoresis: Using Inhomogeneous AC Electrical Fields to Separate and Manipulate Cells. Crit. Rev. Biotechnol..

[10] Shafiee H., Caldwell J.L., Sano M.B., Davalos R.V. (2009). Contactless dielectrophoresis: A new technique for cell manipulation. Biomed. Microdevices.

[11] Wang L., Flanagan L.A., Jeon N.L., Monuki E., Lee A.P. (2007). Dielectrophoresis switching with vertical sidewall electrodes for microfluidic flow cytometry. Lab Chip.

[12] Benhal P., Chase J.G., Gaynor P., Oback B., Wang W. (2014). AC electric field induced dipole-based on-chip 3D cell rotation. Lab Chip.

[13] Ashkin A., Dziedzic J.M. (1971). Optical Levitation by Radiation Pressure. Appl. Phys. Lett..

[14] Ashkin A., Dziedzic J.M. (1987). Optical trapping and manipulation of viruses and bacteria. Science.

[15] Neuman K.C., Block S.M. (2004). Optical Trapping. Rev. Sci. Instrum..

[16] Zhang H., Liu K.K. (2008). Optical Tweezers for single cells. J. R. Soc. Interface.

[17] Kim S.B., Yoon S.Y., Sung H.J., Kim S.S. (2008). Cross-Type Optical Particle Separation in a Microchannel. Anal. Chem..

[18] Guck J., Ananthakrishnan R., Mahmood H., Moon T.J., Cunningham C.C., Kas J. (2002). Stretching biological cells with light. J. Phys. Condens. Matter.

[19] Lincoln B., Schinkinger S., Travis K., Wottawah F., Ebert S., Sauer F., Guck J. (2007). Reconfigurable microfluidic integration of a dual-beam laser trap with biomedical applications. Biomed. Microdevices.

[20] Kolb T., Albert S., Haug M., Whyte G. (2015). Optofluidic rotation of living cells for single-cell tomography. J. Biophotonics.

[21] Dasgupta R., Ahlawat S., Verma R.S., Gupta P.K. (2011). Optical orientation and rotation of trapped red blood cells with Laguerre-Gaussian mode. Opt. Express.

[22] Kreysing M.K., Kiessling T., Fritsch A., Dietrich C., Guck J.R., Käs J.A. (2008). The optical cell rotator. Opt. Express.

[23] Bruus H. (2011). Acoustofluidics 1: Governing equations in microfluidics. Lab Chip.

[24] Johnson D., Feke D. (1995). Methodology for fractionating suspended particles using ultrasonic standing wave and divided flow fields. Sep. Technol..

[25] Yasuda K., Umemura S., Takeda K. (1995). Concentration and fractionation of small particles in liquid by ultrasound. Jpn. J. Appl. Phys..

[26] Hawkes J.J., Coakley W.T. (2001). Force field particle filter, combining ultrasound standing waves and laminar flow. Sens. Actuators B.

[27] Petersson F., Nilsson A., Holm C., Jönsson H., Laurell T. (2004). Separation of lipids from blood utilizing ultrasonic standing waves in microfluidic channels. Analyst.

[28] Petersson F., Nilsson A., Holm C., Jönsson H., Laurell T. (2005). Continuous separation of lipid particles from erythrocytes by means of laminar flow and acoustic standing wave forces. Lab Chip.

[29] Khoury M., Barnkob R., Laub Busk L., Tidemand-Lichtenberg P., Bruus H., Berg-Sørensen K. (2012). Optical stretching on chip with acoustophoretic prefocusing. Proc. SPIE.

[30] Li S., Ren L., Huang P.H., Yao X., Cuento R.A., McCoy J.P., Cameron G.E., Levine S.J., Huang T.J. (2016). Acoustofluidic Transfer of Inflammatory Cells from Human Sputum Samples. Anal. Chem..

[31] Ahmed D., Ozcelik A., Bojanala N., Nama N., Upadhyay A., Chen Y., Hanna-Rose W., Huang T.J. (2016). Rotational manipulation of single cells and organisms using acoustic waves. Nat. Commun..

[32] Pamme N. (2006). Magnetism and microfluidics. Lab Chip.

[33] Megias-Alguacil D. (2013). Surface rotation of liquid droplets under a simple shear flow: Experimental observation in 3D. Soft Mater..

[34] Lednev V.V. (1991). Possible mechanism for the influence of weak magnetic fields on biological systems. Bioelectromagnetics.

[35] Hejazian M., Nguyen N.T. (2016). Magnetofluidic concentration and separation of non-magnetic particles using two magnet arrays. Biomicrofluidics.

[36] Tanyeri M., Johnson-Chavarria E.M., Schroeder C.M. (2010). Hydrodynamic trap for single particles and cells. Appl. Phys. Lett..

[37] Sipos O., Nagy K., Di Leonardo R., Galajda P. (2015). Hydrodynamic Trapping of Swimming Bacteria by Convex Walls. Phys. Rev. Lett..

[38] Shelby J.P., Chiu D.T. (2004). Controlled rotation of biological micro- and nano-particles in microvortices. Lab Chip.

[39] Lim D.S.W., Shelby J.P., Kuo J.S., Chiu D.T. (2003). Dynamic formation of ring-shaped patterns of colloidal particles in microfluidic systems. Appl. Phys. Lett..

[40] Hagiwara M., Kawahara T., Arai F. (2012). Local streamline generation by mechanical oscillation in a microfluidic chip for noncontact cell manipulations. Appl. Phys. Lett..

[41] Lutz B.R., Chen J., Schwartz D.T. (2006). Characterizing Homogeneous Chemistry Using Well-Mixed Microeddies. Anal. Chem..

[42] Bruus H. (2008). Theoretical Microfluidics (Oxford Master Series in Physics).

[43] Zhang J., Yan S., Yuan D., Alici G., Nguyen N.T., Warkianic M.E., Li W. (2016). Fundamentals and applications of inertial microfluidics: A review. Lab Chip.

[44] Di Carlo D. (2009). Inertial Microfluidics. Lab Chip.

[45] Nguyen N.T., Wereley S.T. (2002). Fundamental and Applications of Microfluidics.

[46] Feng Y., Kleinstreuer C. (2013). Analysis of non-spherical particle transport in complex internal shear flows. Phys. Fluids.

[47] Jefferey J.B. (1922). The motion of ellipsoidal particles immersed in a viscous fluid. Proc. Royal Soc. A.

[48] Goldstein H., Poole C.P., Safko J. (2001). Classical Mechanics.

[49] Alberts B., Bray D., Hopkin K., Johnson A.D., Lewis J., Raff M., Roberts K., Walter P. (2013). Essential Cell Biology.

